# Adaptive elevator kinematics optimization based dual response algorithm for determining proper levels in plaster milling process parameters

**DOI:** 10.1038/s41598-023-35119-2

**Published:** 2023-05-31

**Authors:** Pongchanun Luangpaiboon, Sirirat Juttijudata

**Affiliations:** 1 Thammasat University Research Unit in Industrial Statistics and Operational Research, Faculty of Engineering, Thammasat School of Engineering, Thammasat University, Pathumthani, 12120 Thailand; 2 Industrial and Production Management Research Unit, Faculty of Engineering at Sriracha, Kasetsart Univeristy Sriracha Campus, Chonburi, 20230 Thailand

**Keywords:** Engineering, Chemical engineering

## Abstract

This study proposes a novel hybrid approach, called Adaptive/Elevator Kinematics Optimization algorithm based on dual response algorithm (A/EKO-DRA), to enhance the robust parameters estimation and design of the plaster milling process. The A/EKO-DRA method reduces variability while maintaining the desired output target, thereby minimizing the impact of variance on the expected stucco combined water. The performance of the A/EKO-DRA is compared with conventional processes through numerical examples and simulations. The results show that the A/EKO-DRA method has the lowest mean absolute errors among other methods in terms of parameter estimation, and it achieves the response mean of 5.927 percent, which meets the target value of 5.9 percent for industrial enclosures, with much reduction in the response variance. Overall, the A/EKO-DRA method is a promising approach for optimizing the plaster milling process parameters.

## Introduction

Plaster is a cement that is also known chemically as calcium sulfate hemihydrates. It is used in a range of applications such as construction materials, ceramic industries in dentistry, metal casting, jewelry, medical equipment, and cosmetics. The key attribute that makes it popular is its capacity to harden the shape of the mold used with only a little change in size. Plaster is manufactured by heating the naturally occurring material gypsum. This causes some of the moisture in the molecule to evaporate and the molecule's chemical structure changes into plaster or stucco. The usage of calcification machinery, which can be utilized in the production of ceiling panels or gypsum board, is an important aspect of the process for gypsum ore processing. The usage of calcification machinery, which can be utilized in the production of ceiling panels or gypsum board, is an important aspect of the process for gypsum ore processing. In the manufacturing process, crusher machines, conveyors, grinding rolls, and kettle machines are all used.

The newly installed production line also includes a new plaster milling machine that includes the grinding and calcining equipment. This adds to the complexity of the production process control system. With rigorous environmental control, soluble anhydride levels may be kept substantially below their respective acceptable ranges of 5.5 and 6.2 percent, and quicklime can be completely eradicated^1^. The operation of independent factors that occur throughout the plaster milling process, such as mill outlet temperature, air speed in the system, heating in the system (burner output), and other variables, must be controlled.

Quality control is crucial in this manufacturing process because it impacts the system response of stucco mixed water^2^. As a result, quality control and production control can be complex and time-consuming, causing production costs to grow. Previous quality inspection data revealed that the loss was due by low-quality cement manufacturing^3^. To start a new production process, low-quality cement must be removed from the silo. As a result, variable control in the manufacturing process is crucial for the quality of the finished mortar. This sparked the researcher's curiosity in learning more about the components that influence the plaster milling process.

In order to build models and assess issues, the response surface method (RSM) integrates mathematical and statistical methodologies^4,5^. The experimental design plan must determine the level of factors that will provide the best response value^6–8^. In the case of a response that is associated with multiple independent factors or variables. In reality, the correlation model must be estimated because practices frequently lack knowledge of the genuine relationship between the response variable and the independent variable^9^.

Following that, the dual response surface technique involves establishing the ideal setting condition for the controllable parameters in order to reduce performance variability and departure from the decision maker's desired target^10^. This method extends Myers and Carter's basic ridge analysis procedure. To do this, three main strategies are used: experimental design, regression fitting, and optimization. The least squares method is often used for regression fitting to create suitable response functions for the process mean and variance, provided that the data is normally distributed^11^.

However, when dealing with the tradeoffs between the bias and variance components of mean squared error (MSE) in noisy situations, this technique has several drawbacks. When finding optimum setting circumstances, this paper takes the accuracy of the predicted response into serious consideration. Metaheuristics are considered as a component in the construction of various models. The metaheuristic method evolved from the development and adaptation of heuristic methods. The purpose is to enable flexibility in finding answers to any complicated choice problem with many factors, allowing for quick and effective decision making. Even if the outcome does not guarantee an optimal solution, it is satisfactory and allows a search to be completed in a reasonable amount of time.

In today's data-driven enterprises, an automated data system collects and organizes information. Data analysts are frequently overwhelmed by the volume of interlocking data sets acquired to obtain information on all parameters and their interactions, in addition to the standard designed trials^12^. When establishing the optimum setting settings, this paper takes the accuracy of the predicted response into serious consideration. As a result of this research, a fresh technique or metaheuristic evolutionary elements on a dual response algorithm^13^ are introduced. An adaptive elevator kinematics optimization technique is based on the similarities between the elevator kinematics performance method and the solution to challenging problems with optimization. It is simple to implement and can readily overcome various limitations in the dual response method, yielding ever-better outcomes using simple algorithmic principles^14,15^.

Today's highly competitive world necessitates concise, appropriate, and cost-effective supply chain management of items or installations, which in turn necessitates challenging engineering jobs. Engineers must conduct study in order to employ ideas and methods to optimize it. The problem must be mathematically modelled, and computer approaches must be the primary emphasis of the study. This makes optimization tactics vital and interesting to researchers. When the optimum value problem is complicated, multimodal, and has several ideal points, as well as significant non-linearity in the search space, the standard optimization paradigm fails to locate an overall optimal point due to the stagnation of local optima. As a result, there was growing interest in metaheuristic approaches. In metaheuristics algorithms, optimizations are viewed as black boxes with no mathematical modeling requirements^16^.

Metaheuristic algorithms can be used to solve problems in a variety of industries because of their great flexibility and more local optimal avoidance. Artificial intelligence algorithms are classified into several categories in the literature^17^. The idea stems from imitations of natural phenomena's rules as well as their unpredictability. The two main categories are determined by algorithm inspiration in each phase of optimization and the number of randomly generated applicants. The first integrates intelligence, evolution, and physics-based swarm algorithms^18–22^.

The second category includes algorithms that use individuals and populations to develop and enhance at random during the optimization process. There is no algorithm that can successfully address all optimization difficulties. Theoretical literary research may be thought to improve and hybridize with other metaheuristic components. The elevator optimization approach is proposed in this paper with various evolutionary components^23^. Researchers searched additional mechanisms to implement mathematical or random approaches to improve performance^24–26^.

The major purpose of this effort was to establish the best levels of process parameters for a plaster milling process. A conventional factorial design was utilized in the studies, as well as multiple linear regression on mean and standard deviation. An analytical model for optimizing process parameters was established based on the CN or Copeland and Nelson's method^27^. The process parameters investigated in this study were mill outlet temperature, motor fans #1 and #2, burner rate, and classifier. We propose an adaptive elevator kinematics optimization algorithm for developing alternative regression models for the traditional ones based on mean and standard deviation to alleviate the issues associated with the CN described model. The proposed approach has the key advantage of not requiring any fresh series of experimental designs if the model or regression coefficients have no significant effects. The plaster milling process (PMP) is briefly detailed in the second part. The adaptive elevator kinematics optimization technique is described in detail in the section "Proposed method: adaptive elevator kinematics optimization based dual response algorithm (A/EKO-DRA)". The results achieved by the Adaptive Elevator Kinematics Optimization-based Dual Response Algorithm are reported in the section "Numerical results and analysis". Finally, the section "Conclusions and discussions" contains the research's conclusions and discussions, as well as ideas for further research.

## Plaster milling process (PMP)

Typical plaster milling processes start with crushed gypsum from a crusher machine that meets standards for rock size, gypsum moisture, and gypsum purity. The gypsum is then transported from the silo to the new plaster grinding (calcination) process through a chain feeder. The new proposed plaster milling process (PMP) combines crushing and calcination operations with dual motor fans and is designed as a flash calciner manufacturing unit. This PMP will fully grind the gypsum before burning it into a plaster or stucco.

The expert system was used to investigate the plaster milling process, and it was determined that various factors related to the new plaster or stucco milling process, as well as additional conditions for quality control of the system, which sets the product moisture content at 5.5–6.2 percent (the given value has been converted to protect the company's confidentiality).

**Figure 1:**
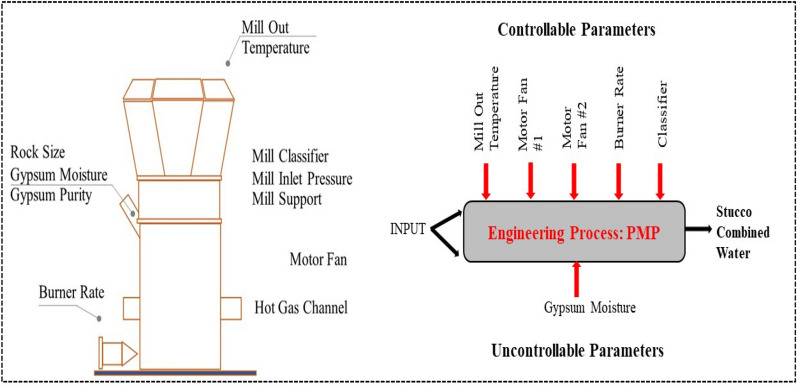
Design and noise parameters of the new PMP.

During this process, several relevant parameters influence the product moisture content (response). The burner rate (%) refers to the controlled heating given to the production process. Classifier (%) is the percentage of the opening of the classifier blade that is used to control the fineness of the mortar. The mill outflow temperature is the temperature at which the plaster and air exit the kiln. Motor Fan #1 and 2 (kW) are the motor power control values utilized to control air circulation in the planned twin motor fan PMP, which are situated at positions 1 (right) and 2 (left). Gypsum moisture is the uncontrollable parameter of the moisture content of gypsum minerals utilized in production (see Fig. 1). The finished stucco will be delivered by air to the dust collector and passed through a cooler drum. It will be moved to the stucco silo where it will be used in the final manufacturing process.

Following the processing of the plaster, a moisture analyzer is utilized to measure the response of Stucco Combined Water with on-the-spot monitoring. The Standard Operating Procedure (SOP) oversees the procedures for collecting mortar samples for use in moisture assessment, ensuring that every worker adheres to the same standards.

## Proposed method: adaptive elevator kinematics optimization based dual response algorithm (A/EKO-DRA)

The dual response algorithm (DRA) for response mean ${\widehat{y}}_{\mu }$ and variance ${\widehat{y}}_{{\sigma }^{2}}$ can be used in robust parameter design for the larger-the-better, smaller-the-better, and nominal-the-best criteria^28^. With the aid of a Factorial Experiment, the impact of various factors and their interactions can be studied. There are two types of effects: the main effect or the effect created by every factor, and interactions with two or more factors or the effects caused by considering factors concurrently. It also makes use of multiple regression analysis to investigate the relationship between independent ${x}_{i}$ and dependent variables ${y}_{\mu }$ and ${y}_{{\sigma }^{2}}$ resulting in prediction models for ${\widehat{y}}_{\mu }$ and ${\widehat{y}}_{{\sigma }^{2}}$ that can be used latter on for the robust process optimization.

1 $${\widehat{\text{y}}}_{{\sigma }^{2}} \, or \, {\widehat{\text{y}}}_{\mu}=\widehat{{\beta }_{0}}+ \sum_{i=1}^{k}\widehat{{\beta }_{i}}{x}_{i} \, {\text{or}}$$

2 $${\widehat{\text{y}}}_{{\sigma }^{2}}\, {\text{or}} \, {\widehat{\text{y}}}_{\mu}=\widehat{{\beta }_{0}}+ \sum_{i=1}^{k}\widehat{{\beta }_{i}}{x}_{i}+\sum_{i=1}^{k}\sum_{j>i}^{k}\widehat{{\beta }_{ij}}{x}_{i}{x}_{j}+\sum_{i=1}^{k}\widehat{{\beta }_{ii}}{x}_{i}^{2}$$

Regression coefficients ${\widehat{\beta }}_{i}$ are normally calculated using least-squares error analysis, however they can alternatively be calculated using various optimization approaches to minimize any selected error indicator. To increase the DRA robustness, the regression coefficients along with experimental data points to be selected for the parameter estimation are determined by Adaptive Elevator Kinematics Optimization (A/EKO) via mean absolute error (MAE) minimization (discussed in later sections). The proposed method is hence called Adaptive Elevator Kinematics Optimization based Dual Response Algorithm (A/EKO-DRA).

In this study, the nominal-the-best scenario is used for DRA robust parameter design. CN or Copeland and Nelson's approach^27^ is adopted i.e. minimizing the response variance $\left({\widehat{y}}_{{\sigma }^{2}}\right)$ while constraining the response mean $\left({\widehat{y}}_{\mu }\right)$ and the controllable variable $\left({x}_{i}\right)$ to be within specific range from the target value and bound, respectively. As a result, the robust parameter design problem for the process becomes:

3 $$\underset{{x}_{i}}{\mathrm{min}}{\widehat{\text{y}}}_{\sigma}$$

subject to

4 $$\left(T-\Delta \right)\le {\widehat{\text{y}}}_{\mu}\le (T+\Delta )$$

5 $$LB\le {x}_{i}\le UB; i =1, 2, \dots , k,$$

where $\Delta$ is the maximum allowable deviation for the response mean (${\widehat{\text{y}}}_{\mu}$) from the specified target value ($T$).

**Figure 2:**
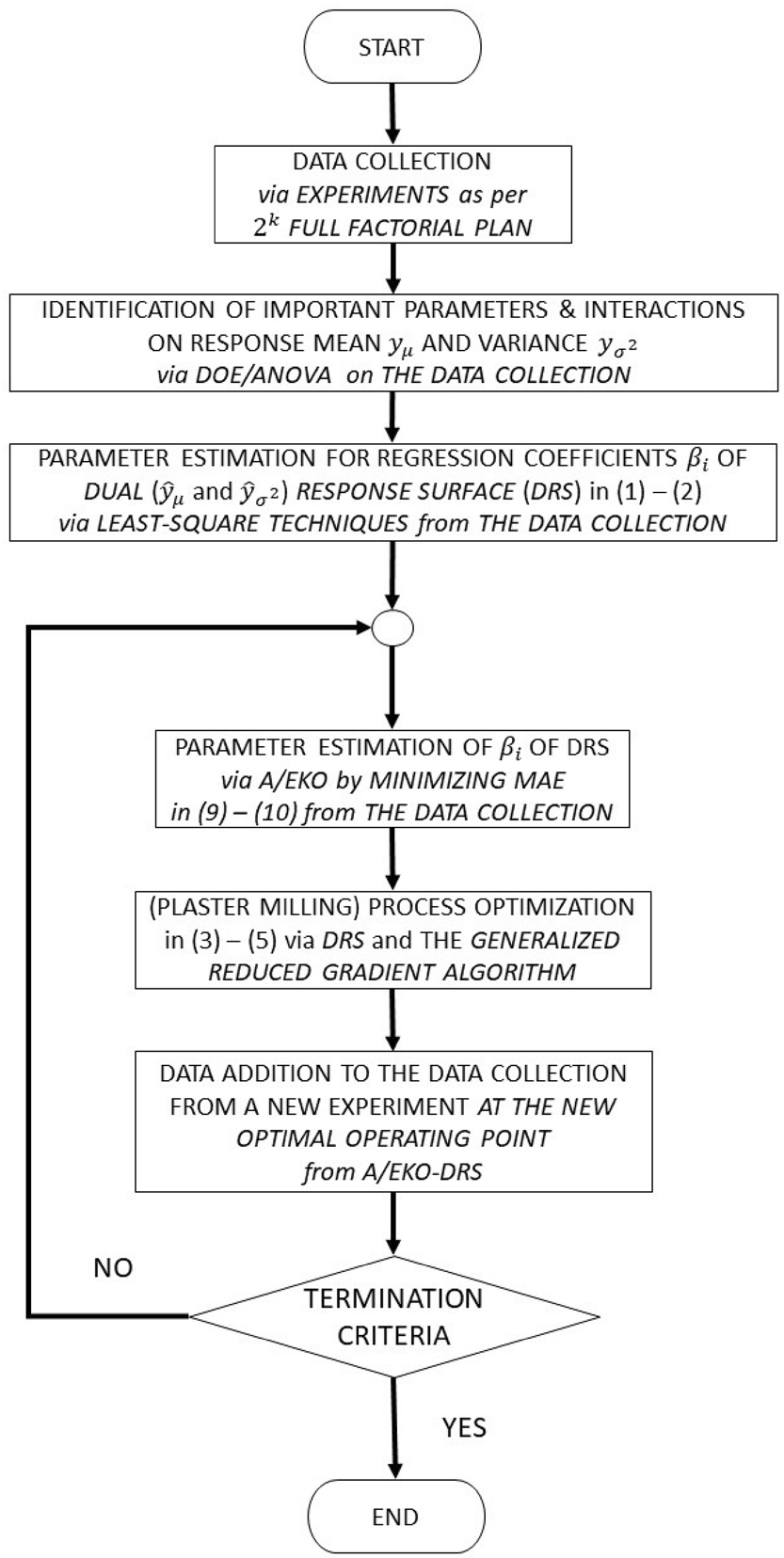
A/EKO-DRA diagram for process improvement.

The robust parameter design is updated iteratively by updating the dual response surface (DRS) multiple regression models in (1)–(2) with additional experimental data done at the optimal parameter set obtained in the previous iteration from (3) to (5) as depicted in Fig. 2.

### Elevator kinematics optimization algorithm

Elevator designs frequently include the associated element in the number of building floors since it influences negotiation prices and mechanical elevator systems. Size, breadth, and height parameters are used to evaluate the motor size and speed of the lift body. Several large corporations are currently competing for the control system factor. The elevator group's control unit is a system that can always monitor the status of elevators via the Internet and online platforms. It seeks to display consumption, status, and data utilization for analysis in elevator targets.

Elevators typically move limited passengers based on their load capacity. As a result, in many large buildings, the elevator waiting time is kept as short as possible in order to maximize passenger comfort. It may also go as quickly as possible to the desired floor while maintaining maximum energy efficiency. Furthermore, the parked floor of elevators must be established in order to meet the needs of the majority of passengers during each usage period. However, the elevator's usage is unidirectional in every phase, which means that if the elevator climbs upstairs, the floor with the calling command of the hall will only be viewed as heading upwards and downwards. This process is repeated until it reaches the maximum level under control. The elevator returns to idle, receives the next order to enter the system, and repeats the process.

Nowadays, manufacturers create different techniques to adjust the degree of control by manipulating the controls into a group structure for the elevator movement to be easily regulated. It can adapt to passenger usage and save energy while moving. It spurred the creation of an elevator kinematics optimization algorithm (EKO) that used the concept of elevator passenger kinetics as a major component in many aspects indicated by the implementation of various elevator groups^29^.

When comparing the EKO structure to the nature of the elevator, it seeks the optimal park floor indicated by the greatest degree of passenger satisfaction^30^. Similarly, optimization approaches are determined in the global optimum by objective function assessment. A passenger command estimate moves the elevator to a different floor. Controlling the elevator group based on distance and time as assessed by proper elevator kinematics functions can change the elevator's position. Iteration can also be used to improve the evaluation of the objective function. Mechanical characteristics as an elevator include machine parameters like as speed, acceleration, and jerk.

Functional improvement is a key solution to process development. The passenger's overall time to reach the target floor may be determined by the distance convergence rate. Lift travel's primary goal is to reduce journey time with fewer pauses in order to improve customer satisfaction and reduce lifting distance. During elevator travel, the same targeted passengers select a level from a possible range at the same time, and those floors are evaluated as a single control. If this floor is frequently used, the memory of floor selection will save the data. This allows the parking regulations on this floor more leeway.

To give a single solution to the optimization problem, each initial answer is likewise chosen at random from a set of values^31^. If the right option is supplied to all of the decision variables' values, that experience is stored in the memory of each variable. There is a good probability that a superior solution will be identified. Elevator memory sizes or the number of elevator memory ($EM$) solutions, speed ($v$), acceleration ($a$), trip height interval ($THI$), the probability of selecting a floor ($PSF$), probability for a reject command ($PRC$), and maximum replication/search iteration ($N$) are all EKO settings.

The distance ($d$) is defined as the absolute difference between the maximin and minimax variables. This distance is used to select one of A, B, or C's three moving requirements (see Table 1). The first is a movement that does not accelerate the transition. A lift does not reach the end of the transitional acceleration, which is the second move or motion to a transitional acceleration. When there are multiple elevator control circumstances, the last move is made.

**Table 1:** New position classified by type or distance of motion.

Motion type	Distance	New position	
A	$d\le \frac{{V}_{1}^{2}}{a}$	$New {X}_{i}={X}_{i}+Rand\left(-\mathrm{1,1}\right)\times \frac{\frac{{V}_{1}^{2}}{2a}}{THI}$	(6)
B	$\frac{{V}_{1}^{2}}{a}\le d\le \frac{{V}_{Max}^{2}}{a}$	$New {X}_{i}={X}_{i}+Rand(-\mathrm{1,1})\times \left(\left(\frac{1}{3a}\right)\left(\frac{{V}_{Max}^{3}}{{V}_{1}}-{V}_{1}^{2}\right)+\frac{{V}_{1}^{2}}{2a} \right)/THI$	(7)
C	$d \ge \frac{{V}_{Max}^{2}}{a}$	$New {X}_{i}= {X}_{i}+Rand\left(-\mathrm{1,1}\right)\times \left(\left(\frac{1}{3a}\right)\left(\frac{{V}_{Max}^{3}}{{V}_{1}}-{V}_{1}^{2}\right)+\frac{{V}_{1}^{2}}{2a}+\left({X}_{b}-{X}_{g}\right)\right)$ where ${X}_{g}$ is the global best solution, ${X}_{b}$ is the best solution at the current position	(8)

Furthermore, while EKO is a resilient strategy for handling hard combinatorial problems and may converge to the optimal solution given enough computation time, it suffers from premature convergence and takes a long time to find high-quality solutions. Solution diversity is a key aspect in enhancing metaheuristic algorithm performance.

This work applies the EKO to two adaptive mechanisms (AM) based Energy consumption in an elevator system: *a revisiting avoidance mechanism based on unnecessary stops or a tabu-list and the intelligent integration of two neighbouring elevators* (AM1) and *adaptive elevator group control* (AM2). The proposed new EKO will be referred to as adaptive elevator kinematics optimization (A/EKO).

Since hydraulic elevators have been phased out, all current elevator systems placed in buildings can be depicted as a counterweight plus cabin and ropes system^32^. This situation can be used to calculate the energy consumption of an elevator hoisting mechanism and its implications. It is possible to conclude that elevators do not expend energy with every movement^33^. In fact, when an elevator goes downwards with less than half the maximum allowable load or upwards with more than half the maximum allowable load, the hoisting system wastes energy.

### Unnecessary stops and neighbourhood (AM1)

The hoisting system, on the other hand, gains energy anytime the deck moves downward with more than half the maximum allowable load or upward with less than half the maximum allowable load. Current brakes use resistors that can recover the energy gained, however not all of the energy can be recovered owing to mechanical friction. Efficiency in dispatching has a significant impact on energy system consumption in this case. It is a common practice to use a policy to avoid unnecessary stops.

When dispatching for average waiting time optimization, it is common to use a policy to avoid unnecessary stops, such as when it is predicted that there will not be sufficient room for all passengers making the landing call, necessitating another stop in the future to collect the passengers who have been left. As a result, instead of making one stop, the elevator would make two, lowering overall performance, especially during peak traffic periods. However, having two stations or neighborhoods instead of one could be profitable in terms of energy. Everything is dependent on the current scenario. In a variety of ways, neighborhood structures can assist the generation of new solutions, allowing for more diversified solutions in the pursuit of an optimal solution.

A neighboring solution is generated by the aforementioned neighbourhood structure. For each acceptable neighboring perturbation, candidates may be deleted from prior solutions^34^. As we all know, if a new generated neighboring solution outperforms the old one in each iteration of A/EKO, the new generated neighboring solution will be accepted. If the new generated neighboring candidate has a worse objective function value than the old one, depending on the present EKO settings and the objective function difference, the new candidate has a probability of being accepted. The prior candidate who was removed from the solution list will be added to the unnecessary stops after the new candidate is accepted. If the unnecessary stop is already full, the candidate that was added to it first will be removed. Candidates on the unnecessary-stop are not allowed to be incorporated into the new solution until it has been eased when we complete the neighbourhood transformation.

### Unpredictable future (AM2)

During the downpeak or uppeak periods, destination or starting floors are often known, and this also occurs during the lunchpeak time (which is a combination of both), limiting dispatching possibilities^35^. Furthermore, because there are so many passengers during these times, waiting time is critical. As a result, the EGCS is usually able to dispatch landing calls during the interfloor pattern, when dispatching options are higher and traffic is lighter (so the waiting time problem is not as important as it is at other times), with the energy problem taking precedence over other factors^36^.

It is difficult to know the destination of each passenger before they reach the cabin, as well as the exact number of passengers who will alight or board on both car and landing calls, without hall call allocation panels on each level. Better detection technologies, such as special cameras, laser beams, or a good mass transducer, can also be employed to detect each passenger as soon as he OR SHE enters the cabin.

If the search process in A/EKO produces the same solution after several iterations, it signifies that the procedure was unable to locate a better solution or escape from this solution. The algorithm will assume that this is a local solution. Figure 2 depicts the process of re-initialization.

### Adaptive elevator kinematics optimization

The dual response used response mean and variance as independent functions for the system under investigation. The functions are then optimized based on the optimization technique used to identify the system's optimum operating conditions. We provide a new optimization technique for a dual response algorithm based on the A/EKO improvement elements in this paper. A traditional DRA is replaced by a series of A/EKO model error minimization in the proposed method.

Vining and Myers^37^ proposed a method for simultaneously optimising mean and variance using Lagrangian multipliers. Lin and Tu^38^ observed that the Vining and Myers approach does not always guarantee global optimum solutions due to the limitation of equality constraints. Based on this, they proposed minimising the mean squared error model by introducing a slight bias to reduce response variability. The minimising MSE function, according to Copeland and Nelson, does not specify how far the estimated mean might deviate from the specified target value. Instead, they altered the VM model by adding a constraint that minimizes the regression model of mean if the squared difference to the target is less than a certain value.

The A/EKO-DRA enhanced dual response algorithm is then proposed to find optimal levels of k influential parameters that contribute to optimal levels of process response. Lower and upper levels of process parameters can also be introduced to prevent design points from extrapolating too far outside the feasible range of the experimental design spaces. The following are the procedure steps for the proposed methodology.

Machine learning metrics are used to assess regression model performance. The most widely used forecast error metrics for point forecasts are mean absolute error (MSE), mean square error (MSE), and root mean squared error (RMSE). The MAE is a commonly used metric because the error value is simple to interpret^39^. Furthermore, MAE error value units are linear and intuitive, and corresponding to the predicted target value unit on the same scale^40^. The integrated expert system with A/EKO methods retains its resilience and exhibits only modest changes when subjected to noise or overstated inputs. Despite the fact that all of these learning metrics are built into the proposed methods in the real process, the fitness function in this study is solely determined by minimizing the mean absolute error (MAE) between the observed (${\widehat{y}}_{\mu }^{Act}\left(i\right)$ or ${\widehat{y}}_{\sigma }^{Act}\left(i\right)$) and estimated (${\widehat{y}}_{\mu }^{Est}\left(i\right)$ or ${\widehat{y}}_{\sigma }^{Est}\left(i\right)$) values, where *i* is the number of observations; and *N* is the sample size^41^.

9 $$MAE=\frac{1}{N}\sum_{i=1}^{N}\left|({\widehat{y}}_{\mu }^{Act}\left(i\right)-{\widehat{y}}_{\mu }^{Est}\left(i\right))\right| or$$

10 $$MAE=\frac{1}{N}\sum_{i=1}^{N}\left|\left({\widehat{y}}_{\sigma }^{Act}\left(i\right)-{\widehat{y}}_{\sigma }^{Est}\left(i\right)\right)\right|$$

## Numerical results and analysis

### Pre-experiment

Newly designed plaster manufacturing processes, combining the griding and calcinating processes, must assure accuracy in order for the product to serve its intended purpose. The first step is to collect baseline data on anticipated factors that may affect the quality or specification of the stucco by an expert system in each subprocedure. The goal of experimental design and analysis is to screen and identify most important parameters and interactions. Table 2 shows the results of the preceding steps.

**Table 2:** Process variables categorized by subprocedures for an A/EKO-DRA application.

Subprocedure	Variable description	Expert system declaration
After crushing	Rock size with 45–50 mm maximum	No involvement
	Gypsum moisture with 5–7% maximum	Uncontrollable variable
	Gypsum purity with 90–95% minimum	No involvement
Mill outlet	Mill outlet temperature	Controllable variable
Mill inlet press	Mill inlet pressure	No involvement
Burner	Burner rate	Controllable variable
Cooler	Stucco after cooler temp	No involvement
Main System Fan	Motor fan #1	Controllable variable
	Motor fan #2	Controllable variable
Classifier	Percent open	Controllable Variable

The second step was to prepare the experiment in terms of both the instrument used to evaluate the response or stucco combined water and the most cost-effective experimental design plan. It could, however, obtain all study results in terms of main effects and interactions. Because the experiment is a test of the actual manufacturing process, the work must be planned as concisely as possible.

The third step was to carry out the experiment as planned and to adjust the level of factors in the real production process in collaboration with all of the experts. The response level value (Stucco mixed water) must be recorded every hour to see the effect of experimenting with the actual production process. The operational settings were adjusted according to the experimental plan for the next 24 h of the experiment until results were achieved to establish A/EKO procedure.

The fourth step was a preliminary trial to verify the study results ultilizing the expert system prior to the proposed algorithm comparisons. In all treatments, the maximum allowable values of Gypsum's uncontrollable factors of Purity and Moisture were 90–95% and 7%, respectively.

### Proposed algorithm comparisons

**Figure 3:**
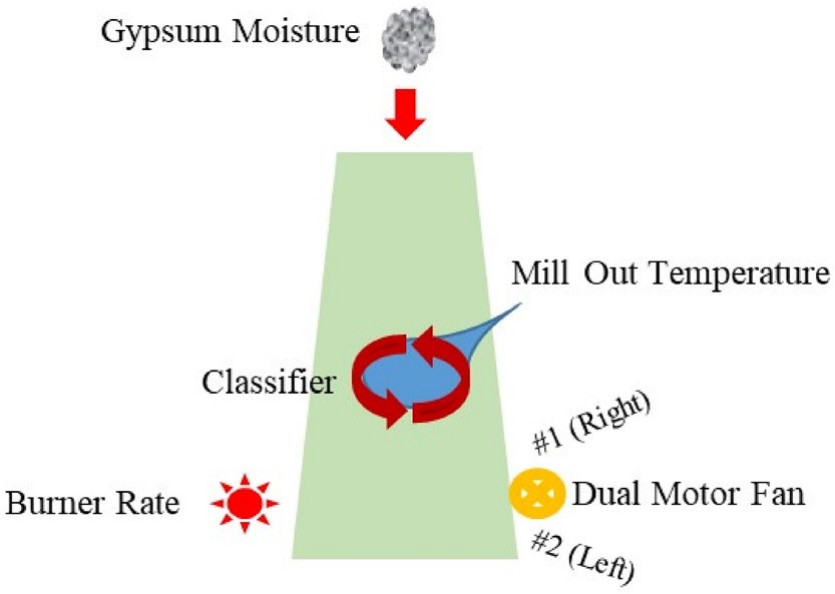
Plaster milling process (PMP).

The engineering department is in charge of measuring the machine's efficiency. The plaster milling process (PMP) with dual motor fans and its main controllable and uncontrollable factors are depicted in Fig. 3.

The numerical results of Metaheuristic evolutionary elements on dual response algorithm are presented in detail in this study. The stucco combined water response has only one response in the PMP ($y$). Explicit constraints keep the parameter levels within their feasible ranges. The A/EKO metaheuristic is used to determine alternate design points in addition to the conventional design point. The A/EKO was constructed based on four influential parameters.

The A/EKO's metaheuristic algorithm optimized the levels of the four influential parameters (${x}_{i}$; *i* = 1, 2, 3, 4). Table 3 shows mill outlet temperature (${x}_{1}$), motor fan #1 (${x}_{2}$), and #2 (${x}_{3}$), burner rate (${x}_{4}$) and classifier (${x}_{5}$) parameters, as well as their current and feasible ranges in coded levels. The following equation can be used to convert a real value (${X}_{i}^{ACTUAL}$) into a coded value (${X}_{i}$) according to the predetermined experimental design:

11 $${X}_{i}={\omega }_{i}( \frac{{X}_{i}^{ACTUAL}-{X}_{i0}^{ACTUAL}}{{\Delta X}_{i}^{ACTUAL}})$$

where ${\Delta X}_{i}^{ACTUAL}$ is the interval between the actual value in the centred point and the real value in the superior or inferior level of a parameter, ${\omega }_{i}$ is the matrix's major coded limit value for parameter, *i*, and ${X}_{i0}^{ACTUAL}$ is the actual value in the centered point. The completely randomized design is adopted to screen all process parameters from all existing design points via the analysis of variance.

**Table 3:** Parameters and their current and feasible coded levels.

Process parameter	Coded level		
	Current	Lower bound (LB)	Upper bound (UB)
${x}_{1}$	0.0005	0.0001	0.001
${x}_{2}$	0.065	0.05	0.075
${x}_{3}$	18	15	20
${x}_{4}$	1	0	2
${x}_{5}$	28	20	30

It began by identifying the number of levels in each parameter using the conventional 2^5^ factorial-design experiments with the requirement that the number of trials be kept to a minimum. The findings of analysis of variance (ANOVA) were employed correctly to summarize the influences arising from the main or all associated interaction effects, as well as the results of model adequacy checking for all normal distribution properties, independence, and constant variance.

The dual-response experiments were designed and analyzed based on the preliminary experimental analysis to assess the influence of various parameters on both the mean and the variance. Before interpreting the analysis of variance, the model's suitability for all properties of normally distributed data, independence, and constant variance must be checked.

A preliminary trial with a two-level five-factor trial design plan and a replicate of two for all treatments was conducted to validate the results obtained through the expert system. However, one additional experiment was performed for some of the methods to determine the factors influencing both the mean and the variance for the proposed method, and the minimum additional run time was attempted. The additional stucco combined water levels for treatments 8, 9, 25, 26, 27, 28, and 29 were 5.96, 5.87, 6.00, 5.92, 5.83, 5.90, and 5.96, respectively. Table 4 shows the experimental results as well as response mean and standard deviation (Stdev).

**Table 4:** The fundamental experimental results were classified according to the treatment for expert system confirmation of the related parameters.

Treatment	Replicate		Statistics for all replicates	
	1	2	Mean	Stdev
1	5.95	5.95	5.948	0.00354
2	5.97	5.95	5.958	0.01768
3	5.96	5.93	5.943	0.02475
4	5.87	5.95	5.908	0.05303
5	5.95	5.95	5.948	0.00354
6	5.95	5.95	5.949	0.00141
7	5.93	5.92	5.926	0.00566
8	5.97	5.95	5.960	0.01000
9	5.94	5.97	5.927	0.05132
10	5.94	5.96	5.950	0.01414
11	5.98	5.88	5.930	0.07071
12	5.94	5.96	5.950	0.01414
13	5.89	5.95	5.920	0.04243
14	6.01	5.93	5.970	0.05657
15	6.00	5.94	5.970	0.04243
16	5.92	5.93	5.925	0.00707
17	5.83	6.00	5.915	0.12021
18	5.90	5.92	5.910	0.01414
19	5.96	5.83	5.895	0.09192
20	5.96	5.90	5.930	0.04243
21	5.97	5.96	5.965	0.00707
22	5.86	5.96	5.910	0.07071
23	5.90	5.87	5.885	0.02121
24	5.90	5.95	5.925	0.03536
25	5.93	5.95	5.960	0.03606
26	6.04	5.93	5.963	0.06658
27	5.96	5.97	5.920	0.07810
28	5.96	5.94	5.933	0.02969
29	5.93	5.94	5.942	0.01662
30	5.93	5.98	5.955	0.03536
31	5.92	5.86	5.889	0.04031
32	5.95	5.90	5.927	0.03748

The experimental results of the ANOVA of mean and variance are shown in Table 5. Only interaction of the mill outlet temperature (${x}_{1}$) with burner rate (${x}_{4}$) and the burner rate (${x}_{4}$) with classifier (${x}_{5}$) influences the response mean at the 95 percent confidence level. The interactions of the mill outlet temperature (${x}_{1}$) with motor fan #1 (${x}_{2}$), motor fan #1 (${x}_{2}$) with motor fan #2 (${x}_{3}$) and motor fan #2 (${x}_{3}$) with classifier (${x}_{5}$), on the other hand, significantly affect the variance response at the same confident interval level. Only the interaction of the motor fan #1 (${x}_{2}$) and burner rate (${x}_{4}$) has a significant influence on both mean and variance responses.

**Table 5:** Analysis of variance (ANOVA) based on response mean and variance.

Source of variation	Mean		Variance	
	F-value	P-value	F-value	P-value
${x}_{1}$	28.90	0.117	1.72	0.415
${x}_{2}$	242.64	0.041	354.91	0.034
${x}_{3}$	2.14	0.382	106.27	0.062
${x}_{4}$	0.04	0.876	85.03	0.069
${x}_{5}$	117.43	0.059	812.70	0.022
${{\varvec{x}}}_{1}$*${{\varvec{x}}}_{2}$	11.82	0.180	164.45	0.050
${x}_{1}$*${x}_{3}$	49.17	0.090	107.39	0.061
${{\varvec{x}}}_{1}$*${{\varvec{x}}}_{4}$	331.51	0.035	2.94	0.336
${x}_{1}$*${x}_{5}$	1.60	0.425	211.75	0.044
${{\varvec{x}}}_{2}$*${{\varvec{x}}}_{3}$	25.97	0.123	332.62	0.035
${{\varvec{x}}}_{2}$*${{\varvec{x}}}_{4}$	173.68	0.048	432.10	0.031
${x}_{2}$*${x}_{5}$	294.49	0.037	73.46	0.074
${x}_{3}$*${x}_{4}$	22.72	0.132	19.72	0.141
${x}_{3}$*${x}_{5}$	23.84	0.129	230.60	0.042
${{\varvec{x}}}_{4}$*${{\varvec{x}}}_{5}$	935.93	0.021	38.73	0.101
${x}_{1}$*${x}_{2}$*${x}_{3}$	37.80	0.103	0.04	0.879
${x}_{1}$*${x}_{2}$*${x}_{4}$	56.04	0.085	295.60	0.037
${x}_{1}$*${x}_{2}$*${x}_{5}$	1.61	0.425	885.25	0.021
${x}_{1}$*${x}_{3}$*${x}_{4}$	16.02	0.156	330.56	0.035
${x}_{1}$*${x}_{3}$*${x}_{5}$	87.41	0.068	269.33	0.039
${x}_{1}$*${x}_{4}$*${x}_{5}$	0.28	0.690	725.26	0.024
${x}_{2}$*${x}_{3}$*${x}_{4}$	60.56	0.081	5.19	0.263
${x}_{2}$*${x}_{4}$*${x}_{5}$	4.67	0.276	306.77	0.036
${x}_{2}$*${x}_{4}$*${x}_{5}$	4.06	0.293	24.94	0.126
${x}_{3}$*${x}_{4}$*${x}_{5}$	40.47	0.099	3.32	0.320
${x}_{1}$*${x}_{2}$*${x}_{3}$*${x}_{4}$	10.58	0.190	585.74	0.026
${x}_{1}$*${x}_{2}$*${x}_{3}$*${x}_{5}$	139.44	0.054	283.59	0.038
${x}_{1}$*${x}_{2}$*${x}_{4}$*${x}_{5}$	94.97	0.065	501.43	0.028
${x}_{1}$*${x}_{3}$*${x}_{4}$*${x}_{5}$	231.81	0.042	90.30	0.067
${x}_{2}$*${x}_{3}$*${x}_{4}$*${x}_{5}$	50.98	0.089	170.63	0.049
${x}_{1}$*${x}_{2}$*${x}_{3}$*${x}_{4}$*${x}_{5}$	331.41	0.035	510.22	0.028

These experimental results were used to confirm the variables involved in the problem of interest. All variables screened by a system of experts working with the cementing engineer to develop the cement production process influence the established response, some of which may have both mean and variance effects, while others may just have mean or variance effects. The experiment will then incorporate a management system that uses a metaheuristic approach to continuously search for optimal parameter levels of factors. The experimental level must be chosen with care in the early stages so that experts can detect errors quickly and efficiently before they are used in the actual production system. A multivariate linear model and the enhanced dual response algorithm with the A/EKO (A/EKO-DRA) were used. The multivariate quadratic model is also taken into accounted in subprocedures included in the process development process.

Apart from the typical experiment design, the next issue is attempting to analyze the data from multiple or disjointed sources. Various types of data are frequently housed in a comprehensive and centralized system. Analysts will have to assess a wide range of information, allowing for cross-comparisons and ensuring that data is complete using multi-step response surface methods. AM, EKO, and A/EKO were built to calculate stucco combined water based on the mill out temperature (${x}_{1}$), motor fan #1 (${x}_{2}$), and #2 (${x}_{3}$), burner rate (${x}_{4}$) and classifier (${x}_{5}$). Visual Basic 2017 on an AMD Ryzen 5 2400G with Radeon Vega Graphics 3.60 GHz PC is used to estimated response models and solve the optimization problem. EKO's optimal parameters are also critical.

The values of $EM, a, v,THI, PRC, PS{F}_{min}$ and $PS{F}_{max}$ in this study were defined as 40, 1, 0.8, 100, 0.95, 0.40 and 0.60, respectively, as per^42^, who thoroughly studied them to find optimal values. Data from previously available operating conditions were used to estimate the dual response surface regression coefficients ${\beta }_{i}$ in (1)–(2). A/EKO combines the capabilities and benefits of both AM and EKO technologies. In each iteration, two best solutions are compared, then better candidates are assigned to the global best solutions. Both algorithms are capable of coping with parameter estimation difficulties. AM and EKO are driven to develop new candidates by transferring system solution parameters to their global best.

If the best EKO solution is influenced by the best AM solution, the improved EKO's motion direction will be examined. As a result, the direction operator must be updated during the A/EKO iteration. Initialization and solution management are the two main algorithms of A/EKO. Table 6 shows the solution handling steps for the AM, EKO, and A/EKO algorithms. The regression coefficients for the response mean (${\widehat{y}}_{\mu }$) and variance (${\widehat{y}}_{{\sigma }^{2}}$) can be iteratively updated until mean absolute error (MAE) for  ${\widehat{y}}_{\mu }$ and ${\widehat{y}}_{{\sigma }^{2}}$ response surfaces are minimal for the given set of data collection by means of A/EKO:

**Table 6:** Estimated coefficients of multiple regression.

Response	Coefficients	Least squares techniques	EKO		
			AM1	AM2	AM
${\widehat{y}}_{\mu }$	$\widehat{{\beta }_{0}}$	− 0.000343	− 0.000650	− 0.000299	− 0.000698
	$\widehat{{\beta }_{1}}$	− 5.528624	− 5.595736	− 5.589455	− 5.601736
	$\widehat{{\beta }_{2}}$	0.672766	0.668565	0.676981	0.662034
	$\widehat{{\beta }_{3}}$	− 0.000525	− 0.000568	− 0.000369	− 0.000429
	$\widehat{{\beta }_{4}}$	− 0.002048	− 0.001963	− 0.001857	− 0.001102
	$\widehat{{\beta }_{5}}$	1.697655	1.097655	1.259747	0.857925
	$MAE$	1.697655	1.097655	1.259747	0.857925
${\widehat{y}}_{{\sigma }^{2}}$	$\widehat{{\beta }_{0}}$	− 0.000744	− 0.000685	− 0.000902	− 0.000840
	$\widehat{{\beta }_{1}}$	− 0.409258	− 0.397410	− 0.389577	− 0.381147
	$\widehat{{\beta }_{2}}$	− 0.050336	− 0.038064	− 0.050243	− 0.045282
	$\widehat{{\beta }_{3}}$	0.000214	0.000197	0.000096	0.000102
	$\widehat{{\beta }_{4}}$	− 0.000384	− 0.000288	− 0.000302	− 0.000242
	$\widehat{{\beta }_{5}}$	0.000115	0.000227	0.000089	0.000109
	$MAE$	3.630225	3.287350	3.897230	3.005420

12 $${\widehat{y}}_{\mu }=-0.000698-5.601736{x}_{1}+0.662034{x}_{2}-0.000429{x}_{3}-0.001102{x}_{4}+0.857925{x}_{5}$$

13 $${\widehat{y}}_{{\sigma }^{2}}=-0.000840-0.381147{x}_{1}-0.045282{x}_{2}+0.00102{x}_{3}-0.000242{x}_{4}+0.00109{x}_{5}$$

After obtaining the dual response surface models for ${\widehat{y}}_{\mu }$ and ${\widehat{y}}_{{\sigma }^{2}}$ from A/EKO-DRA using the actual response as a reference^43^, the optimal parameter levels were determined by using Copeland and Nelson’s dual response algorithms in (3)–(5). The maximum deviation ($\Delta$), which is calculated from the response mean (${\widehat{\text{y}}}_{\mu}$), and the specified target value ($T$) are set at 0.05 and 0.059, respectively.

**Figure 4:**
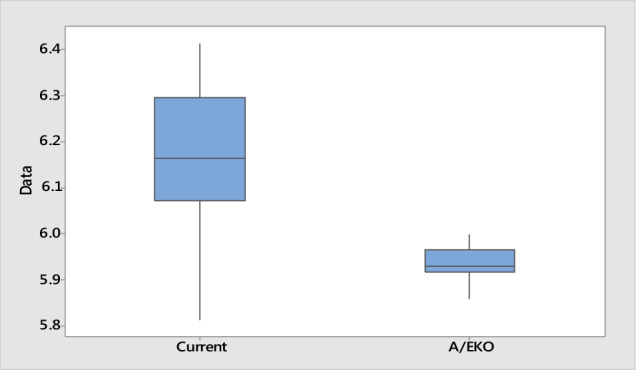
Performance measures of mean and variance from the current and A/EKO operating condition.

Table 6 shows the regression coefficients ${\beta }_{i}$ and relative error predictions for each method. It was evident that the proposed A/EKO method outperforms AM and EKO. Process optimization using A/EKO-DRA optimization yields the optimal values of mill out temperature (${x}_{1}$), motor fan #1 (${x}_{2}$), and #2 (${x}_{3}$), burner rate (${x}_{4}$) and classifier (${x}_{5}$) are at 0.001, 0.05, 15, 0, and 30, respectively (Table 7). Further, it is also observed that the mean of stucco combined water changes from 6.152% to 5.927% based on gypsum purity of 90–95%, while the variance of stucco combined water is statistically reduced from 0.152 to 0.034 (Fig. 4).

**Table 7:** Current and new A/EKO-DRA settings for optimal plaster milling process.

Process parameter	Coded level	
	Current	A/EKO
${x}_{1}$	0.0005	0.001
${x}_{2}$	0.065	0.05
${x}_{3}$	18	15
${x}_{4}$	1	0
${x}_{5}$	28	30

## Conclusions and discussions

This research presents the framework for the adaptive elevator kinematics optimization using the dual-response surface algorithm (A/EKO-DRA) to solve the constraints of the response surface method and make it more appropriate for usage in noisy environment optimizations. To define new solutions without further experimental design implementation or extra design points, one goal is to determine the significant effects of the key parameters.

The technique is used to drive the stucco combined water to reach the industrial standard target with minimal variation amid changing environmental conditions during the plaster milling process. The novel meta-heuristic search method, A/EKO, is integrated to create process settings. The effectiveness of this strategy in combination with the prior operating state is also attempted to be assessed. The results of an experimental investigation show that the number of stucco combined water from the proposed one is, on average, closer to the objective than the previous one. As a result, it increases the likelihood that the proposed method will be efficient as well as robust. Future study in this field ought to be promoted.

## Data Availability

Data available on request from the authors: The data that support the findings of this study are available from the corresponding author, [Sirirat JUTTIJUDATA], upon reasonable request.
